# The Systematic Development and Pilot Randomized Evaluation of Counselling for Alcohol Problems, a Lay Counselor‐Delivered Psychological Treatment for Harmful Drinking in Primary Care in India: The PREMIUM Study

**DOI:** 10.1111/acer.12653

**Published:** 2015-02-19

**Authors:** Abhijit Nadkarni, Richard Velleman, Hamid Dabholkar, Sachin Shinde, Bhargav Bhat, Jim McCambridge, Pratima Murthy, Terry Wilson, Benedict Weobong, Vikram Patel

**Affiliations:** ^1^ London School of Hygiene & Tropical MedicineLondonUnited Kingdom; ^2^ SangathGoaIndia; ^3^ University of BathBathUnited Kingdom; ^4^ ParivartanSataraIndia; ^5^ National Institute of Mental Health & NeurosciencesBangaloreIndia; ^6^ Department of PsychologyRutgers, The State University of New JerseyNew BrunswickNJ; ^7^ Centre for Chronic Conditions & InjuriesPHFINew DelhiIndia

**Keywords:** Harmful Drinking, Lay Counselors, Primary Care, India, Psychological Treatment

## Abstract

**Background:**

Despite harmful drinking causing a significant burden on global health, there is a large treatment gap, especially in low‐ and middle‐income countries. A major barrier to care is the lack of adequately skilled human resources to deliver contextually appropriate treatments. This paper describes the systematic process used to develop Counselling for Alcohol Problems (CAP), a brief psychological treatment (PT) for delivery by lay counselors in routine primary care settings to men with harmful drinking in India.

**Methods:**

CAP was developed using a methodology involving 3 sequential steps: (i) identifying potential treatment strategies; (ii) developing a theoretical framework for the treatment; and (iii) evaluating the acceptability and feasibility of the treatment.

**Results:**

CAP is a 3‐phase treatment delivered over 1 to 4 sessions based on a motivational interviewing (MI) stance and involves the following strategies: assessment and personalized feedback, family engagement, drink refusal skills, skills to address drinking urges, problem‐solving skills and handling difficult emotions, and relapse prevention and management. Data from a case series were used to inform several adaptations to enhance the acceptability of CAP to the recipients and feasibility of delivery by lay counselors of the treatment, for example expansion of the target group to include alcohol‐dependent patients and the extension of the delivery settings to include home‐based delivery. There was preliminary evidence of the effectiveness of CAP.

**Conclusions:**

CAP is an acceptable brief PT for harmful drinking delivered by lay counselors in primary care whose effectiveness is currently being tested in a randomized controlled trial based in primary care in Goa, India.

Alcohol use disorders (AUD), the leading causes of the global burden of disease (Whiteford et al., 2013), cause large costs to societies attributable to health care and social harm (Rehm et al., 2009; WHO Global InfoBase Team, 2005). India, the target of market expansion by producers of alcoholic beverages (Benegal, 2005; Caetano and Laranjeira, 2006), has been experiencing an increase in alcohol availability/consumption, lowering of the age of drinking onset, and higher levels of alcohol‐related problems (Pillai et al., 2014; Prasad, 2009).

AUD range from hazardous drinking, to harmful drinking and ultimately to alcohol dependence (Reid et al., 1999). The World Health Organization (WHO)'s (mhGAP‐Mental Health Gap Action Programme) guidelines advocate the use of brief advice for hazardous drinking and motivational interviewing (MI)‐based brief psychological treatments (PTs) for harmful drinking (World Health Organization, 2010). Among all mental disorders, globally, AUD have the widest treatment gap; the contact coverage of care for AUD is less than 20% in most countries (Kohn et al., 2004). Furthermore, as most patients who are in contact with services do not have their AUD recognized or receive evidence‐based treatments, the “effective” coverage gap is likely to be even larger (De Silva et al., 2014).

Major reasons for this treatment gap are the lack of mental health specialists skilled in delivering PT and the contextual barriers toward delivery of PT in low resource settings (Knapp et al., 2006; World Health Organization, 2005). Task sharing (rational redistribution of tasks among health workforce teams) with nonspecialist health workers is advocated to address such human resource shortages (Lawn et al., 2008; Lewin et al., 2005). Eng and colleagues (1997) have conceptualized nonspecialist health workers on a spectrum from the “natural helper” (unpaid community members) at one end to the “para‐professional” (paid workers with minimal qualifications, trained, and demonstrating acceptable levels of standardized competencies) at the other. Community health workers have demonstrated effectiveness in increasing access to care (Swider, 2002; Viswanathan et al., 2010), for example, promoting immunization uptake and breastfeeding, improving tuberculosis treatment outcomes, and reducing child morbidity and mortality (Lewin et al., 2010). In the field of mental health, there is robust evidence that lay counselors (a person without professional qualification in mental health care) can be trained to deliver PT effectively for people with depressive and anxiety disorders in low‐ and middle‐income countries (LMIC) (van Ginneken et al., 2013). There is also evidence demonstrating that PT developed in Western cultural contexts retain their effectiveness in different cultural contexts, when adapted following a systematic methodology (Chowdhary et al., 2013). A key Grand Challenge in Global Mental Health is to design a methodology for the development and evaluation of PT by lay counselors (Collins et al., 2011).

The purpose of this paper is to define a PT “discovery” process in order to develop a brief PT for harmful drinking to be delivered by lay counselors in routine primary care settings. The treatment was developed to address harmful drinking in men because the vast majority of persons with any kind of AUD in the region were men (Murthy et al., 2010). This work is a part of the PRogrammE for Mental health Interventions in Under‐resourced health systeMs (PREMIUM) whose guiding principles are to develop PT based on both global and contextually relevant evidence and with emphasis on both acceptability to patients and feasibility for delivery by lay counselors in routine healthcare settings (Patel et al., 2014). In PREMIUM, the lay counselors are “para‐professionals” as described by Eng and colleagues. (1997). The fieldwork was carried out in Satara and Goa in India. Goa is a small state on the west coast of India with a population of over 1.4 million; Satara is a semi‐urban district in the state of Maharashtra with a population of over 2.8 million. All research procedures with human participants were approved by the Institutional Review Boards (IRB) of Sangath and the London School of Hygiene & Tropical Medicine, and the Health Ministry Screening Committee of the Indian Council of Medical Research, and written informed consent was obtained from all participants.

## Materials, Methods, and Results

**Figure 1:**
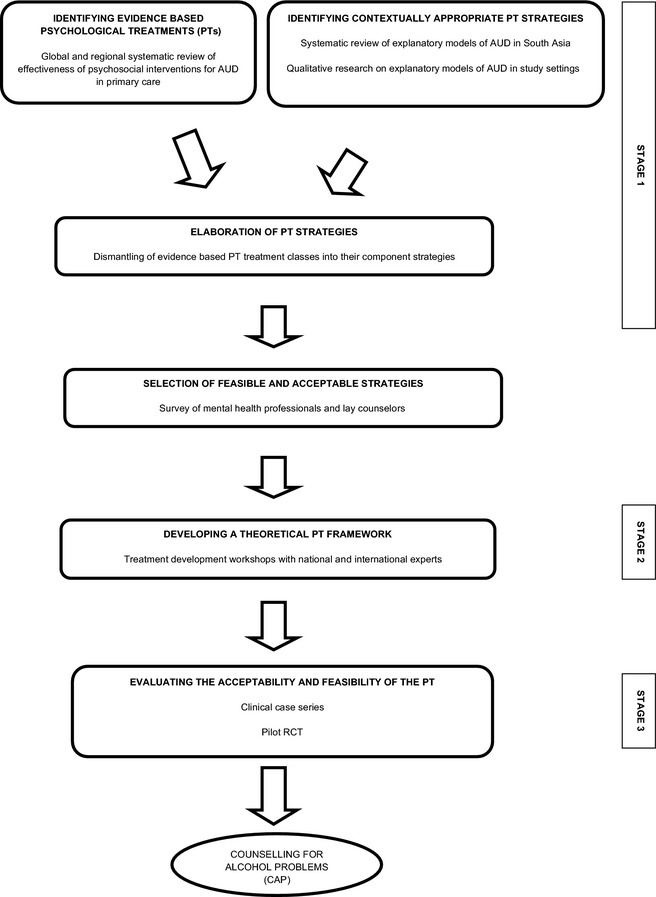
An overview of the process of development of the Counselling for Alcohol Problems.

The framework for the development of Counselling for Alcohol Problems (CAP) was informed by our systematic review of methods used for cultural adaptations of PTs for depression (Chowdhary et al., 2013), the approach of “dismantling” evidence‐based PT to “distill” core treatment strategies which could be more easily task‐shared with less qualified therapists (Chorpita et al., 2005), and the methodology adopted by the research team in India for the development of psychosocial interventions for delivery by lay counselors for other mental disorders (Chatterjee et al., 2003, 2008; Dias et al., 2008). Based on these experiences, our methodology involved 3 sequential stages: (i) identifying potential treatment strategies; (ii) developing a theoretical framework for the treatment; and (iii) evaluating the acceptability, feasibility, and impact of the treatment. Within each stage, several procedures were implemented as shown in Fig. 1.

**Figure 2:**
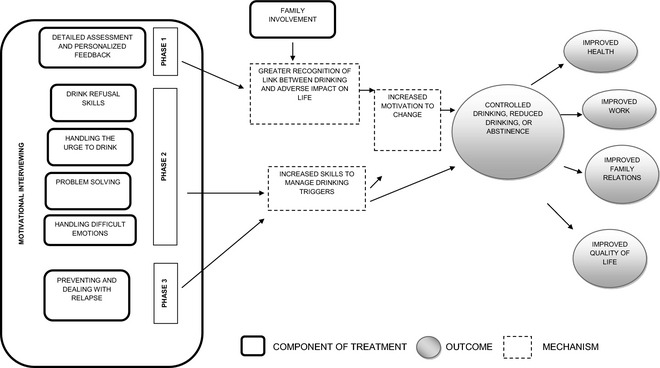
Modeling of the CAP components and pathways to outcome.

Our methodology is the reverse of the traditional approach of using theory to drive the development of a set of coherent treatment strategies. Our primary rationale for adopting this method was to ensure that we were starting the treatment development process by selecting strategies which had been shown to be most effective and appeared to be best suited to be delivered by lay counselors. However, we did not want our treatment to simply be a disparate collection of unrelated individual strategies; thus, we subsequently aimed to knit them together into a coherent, theory‐driven conceptual framework (as shown in Fig. 2) which would provide a rationale for how these strategies led to the ultimate health change we sought. We provide below a detailed description of the methods and the results generated at each stage of treatment development, and in conclusion, we present the conceptual framework of the final treatment (Fig. 2).

### Stage 1: Identifying Potential Treatment Strategies

The goal of this step was to identify promising strategies (drawn from both global and contextually relevant evidence) which could be considered as components of CAP keeping in mind evidence of their acceptability to patients, their effectiveness, and their potential for delivery by lay counselors.

#### Identifying Empirically Supported Treatments

We updated the WHO mhGAP guidelines for evidence‐based interventions for mental disorders in nonspecialized healthcare settings (World Health Organization, 2010) with a literature search on PT for AUD from January 1, 2009, to March 31, 2011, on MEDLINE and PsycINFO and a literature review of regional (South Asian) literature on PT for AUD (details can be accessed online in Appendices S1 and S2). Thirty‐one randomized controlled trials (RCTs) and 8 systematic reviews in the international literature review and 28 studies from South Asia generated a list of treatments which were then rated by the research team and selected for further consideration based on the strength of the evidence of effectiveness and the generalizability of this evidence to the context (Table S1). All treatments recommended by mhGAP were automatically considered for the next stage. The international review supported the use of brief interventions (BI) based on MI, CBT, social norms intervention, and personalized feedback. The regional review supported the use of family therapy, BI, behavioral therapy, 12‐step facilitation, “community‐based complex intervention,” and Kundalini Yoga.

#### Defining Contextually Appropriate Goals and Content of Treatment

It is important to take explanatory models into account while designing a contextually relevant PT as such beliefs and behaviors related to AUD influence help‐seeking behavior, subsequent adherence with treatment, and eventual patient outcomes. This step used the following methods: (i) systematic review of the literature on explanatory models of AUD from South Asia; and (ii) in‐depth interviews (IDI) with patients with harmful drinking and alcohol dependence, and family caregivers, in the 2 study sites in India, and with mental health professionals from various parts of India. Findings from these 2 methods were triangulated to achieve the study aims of this step. The detailed methodology of the IDI has been published elsewhere (Nadkarni et al., 2013). Key findings of the IDI included the following: Internal factors (e.g., stress due to a variety of reasons) as well as external factors (e.g., peer pressure) were perceived to lead to AUD. Men with AUD used a range of strategies to help cope with the problems related to their drinking, for example, getting involved in activities that distract from drinking and in religious/spiritual activities. This data was used in the subsequent steps to support the inclusion of strategies such as “drink refusal skills” (to counter peer pressure) and “handling drinking urges” (to distract from cravings to drink). Table 1 indicates which strategies were supported by data from the IDI.

**Table 1:** Selection of Psychological Treatment Strategies for the Counseling for Alcohol Problems

Treatment strategy	Source					Perceived acceptability, feasibility, effectiveness, and safety
	Literature review		In‐depth interviews			
	International	Regional	Mental health professionals	Patients	Family care givers	
Personalized feedback	✓	✓	✓	✓		++
Motivational interviewing	✓	✓	✓			++
Psychoeducation	✓	✓	✓	✓	✓	++
Supportive counseling	✓	✓	✓		✓	++
Cognitive restructuring	✓	✓	✓			+
Problem solving	✓	✓	✓			++
Enlisting social support	✓	✓		✓	✓	++
Support groups	✓	✓	✓	✓	✓	++
Vocational counseling	✓		✓	✓		++
Relapse prevention	✓	✓	✓	✓		++
Social skills training	✓	✓	✓		✓	++
Family psychoeducation	✓	✓	✓	✓	✓	++
Family counseling	✓	✓	✓	✓	✓	++
Relaxation	✓		✓	✓	✓	++
Geographical cure			✓	✓		++
Physical exercise			✓	✓		++
Religious and spiritual practices	✓	✓	✓	✓		−
Addressing unconscious mechanisms			✓			−
Addressing interpersonal issues with one's partner	✓	✓	✓	✓		−
Focus on past experiences and relationships	✓		✓			−
Music therapy			✓	✓		−

Perceived acceptability, feasibility, effectiveness, and safety: ++ High acceptability, feasibility, perceived effectiveness, and safety; + moderate acceptability, feasibility, and perceived effectiveness; and some safety concerns—not recommended for further consideration.

#### Dismantling Treatments into Component Strategies

We “dismantled” the evidence‐based treatment *classes* (composites of individual strategies*)* identified earlier into their component *strategies*, following a distillation process to identify common strategies of evidence‐based treatments (Chorpita et al., 2005). From the international literature, we only considered strategies derived from treatments which met our criteria on effectiveness and generalizability. To these we added *all* strategies identified from the regional literature and IDI, irrespective of the strength of evidence of effectiveness. Then, the list of strategies was synthesized by merging similar strategies, removing those which were considered to reflect *techniques* (specific procedures used to implement a strategy, e.g., self‐monitoring), and further dismantling some which were composites of discrete strategies. This left a list of 21 separate strategies as shown in Table 1, with the source from where they were derived.

#### Selection of Feasible and Acceptable Strategies

A survey was conducted to identify which of these 21 strategies would be most feasible and safe for delivery by lay counselors and acceptable by patients in the Indian context. The survey was conducted with 2 groups of participants selected by maximum variability sampling: (i) mental health professionals representing a range of geographical regions of the country and professional disciplines, such as psychiatry and clinical psychology; and (ii) lay counselors (as defined previously) from Goa. Fifteen mental health experts (71% response rate) and 43 lay counselors (93% response rate) completed the survey. Of the 21 strategies included in the survey, 16 strategies were carried forward to the next stage from this step.

### Stage 2: Developing a Theoretical Treatment Framework

The goal of this stage was to develop a theoretical framework for the treatment based on the component strategies identified in the previous step. This involved 6 treatment development workshops where Indian mental health experts and international experts assembled the strategies into a coherent treatment and identified a theoretical foundation for the treatment. Ranking, pile sorting, and scheduling methods were employed to finalize the list of strategies, organize them into related groups, and schedule them into a coherent treatment delivery (Bernard, 2002). Thirty Indian and 11 international mental health professionals participated in the workshops.

At this stage, cognitive restructuring, support groups, and family therapy were removed as they were felt to be beyond the abilities of a lay counselor; vocational counseling was removed as it was felt that it would require considerable networking and awareness of the job market of the area; family psychoeducation was merged with family counseling and the resulting strategy referred to as family engagement; and support groups were removed due to perceived low feasibility in primary care and the concerns about privacy/confidentiality. The grouping and scheduling of the delivery of the strategies led to the framework of a treatment with up to 4 sessions. The first session comprised assessment, personalized feedback, and psychoeducation. The next session, targeted at addressing practical problems, consisted of problem‐solving skills and social skills training. The subsequent sessions aimed at strengthening coping and relapse prevention, empowering the client with strategies to deal with the risk of relapse. The experts concurred that MI should be the “core” stance which had the strongest evidence base relevant to the goals of PREMIUM and which would form the foundation upon which the other identified strategies would be delivered. Figure 2 illustrates the various components of CAP and the potential mechanisms through which they lead to the eventual treatment goals. CAP is built around a core foundation of MI upon which the various other strategies are delivered. Thus, MI techniques are used across all the sessions to help patients develop and maintain their motivation to change.

The Project MATCH MET manual (Miller et al., 1994), Project MATCH CBT manual (Kadden et al., 1995), COMBINE manual (Zarkin et al., 2008), and Helping Patients Who Drink Too Much: A Clinician's Guide (National Institute on Alcohol Abuse and Alcoholism, 2005) were then identified in consultation with the experts as potential starting points for the development of the CAP manual. Each manual was evaluated by assessing the adequacy of coverage of selected strategies, its suitability for use by lay counselors, and the extent of adaptations needed for the local context, and based on this process, the Project MATCH MET manual was selected for the next stage.

### Stage 3: Evaluating the Acceptability, Feasibility, and Impact of the Treatment

Using the Project MATCH manual as a starting point, the goal of this stage was to develop the CAP manual by adding contextually relevant strategies. The structure and procedures used to deliver this treatment were constantly tested and refined in order to optimize CAP's acceptability and feasibility, first through a case series, followed by an evaluation in a pilot RCT.

#### Evaluating the Acceptability and Feasibility of the Treatment: Case Series

We carried out a clinical case series in which the treatment was delivered by first (i) local mental health professionals (2 psychiatrists, 1 psychiatric social worker, and 1 experienced lay counselor who had been closely involved in the previous stages of the treatment development process) who were supervised by a clinical psychologist with 3 decades experience of counseling in the field of addictions (RV) and subsequently (ii) 19 lay counselors, recruited from the local community and trained and supervised by the local mental health professionals, based in 11 primary healthcare centers (PHC) in Goa. Further details of the recruitment, training, and competency of the lay counselors are published elsewhere (Patel et al., 2014; Singla et al., 2014) and briefly summarized here. Lay counselors were recruited through advertisements in newspapers and a local television channel. Applicants who had completed at least tenth grade education and were fluent in local languages were selected for the interview. Of the 188 applicants, 128 were selected for the interview. The interview involved answering a structured questionnaire, a brief role play to test for skills such as empathy and questions to evaluate willingness to be part of a team, communication, and interpersonal skills. Following the interview, 31 candidates were invited for and completed the training. Of these 31, 20 were selected for the internship in the case series described above. One of the lay counselors dropped out shortly before the internship. During the internship, the lay counselors delivered CAP to patients in PHC and were supervised in groups by experts drawn from the group of local mental health professionals who undertook the first part of the clinical case series. CAP was iteratively revised based on observations made continuously through the case series.

Participants were identified through screening of PHC attenders or were referrals from general practitioners and psychiatrists or were self‐referred clients. Males aged 19 years and above who had a clinical diagnosis of AUD from a mental health professional or who scored 12+ on the Alcohol Use Disorders Identification Test (AUDIT), an internationally validated screening questionnaire (Saunders et al., 1993), were eligible for the case series. Data were collected by independent researchers during the case series and at 1 to 4 months postrecruitment. A range of data were collected on the clinical process, for example, on the engagement of patients, the ease of use of specific strategies and modifications made, and structural barriers in the delivery of the treatment. Two‐hundred forty‐one patients were recruited, 37 receiving treatment from the mental health professionals in the first part of the case series and 204 from the lay counselors in the second part. The data sources were the clinical session notes, 6 serial focus group discussions (FGDs) with the lay counselors, and semi‐structured interviews with 32 purposively sampled patients (10 dropouts, 22 in treatment or treatment completers). All interviews and FGDs were audiotaped; the recorded interviews were transcribed verbatim, and local language interviews were translated into English. The broad themes used for the interviews and group discussions provided the a priori framework, and codes were derived for each of them inductively from the data. Transcripts were then coded in Nvivo 8 (QSR International Pty Ltd, Victoria, Australia). Data from all sources were triangulated to address key questions regarding the acceptability and feasibility of the CAP.

Patients expressed satisfaction with the treatment and reported being pleased at their reduced drinking and the resultant positive impact on their life. They endorsed how the counseling helped them understand their drinking and its effect on their health and techniques that they learned to manage their drinking (Box 1).

Box 1 **Acceptability and Feasibility of CAP**

**Acceptability to Patients**

“I used to spend a lot of money on drink every month. But now all that money is getting saved; in last two months I have saved Rs. 5,000 to 6,000, which I would have spent on drinks. With the help of the counsellor, I have stopped drinking completely since last 2 months. After work, I used to go directly to the bar but now I go directly home and pray to god. Now I spend my evenings in the library and playing with my kids.” (Male, 30 years)

**Acceptability to Lay Counselors**

“In the beginning week, I was too scared. I simply had no idea on what to do and how to do (Counseling).” Though we have had role‐plays during the training programme, our confidence level was low. Once we started working in PHC, every single day was a new experience; a new learning. I used to take a lot of time in asking questions and patients were not ready to wait. I had difficulty in understanding what to and how to ask. But gradually with the support of the clinic supervisors and through the supervision in the weekly meeting, I gained confidence and developed my own style (of counseling).” (Female, 22 years)

**Barriers to Accessing Psychological Treatments**

“…the main reason for not completing the session is I work as a truck driver. I am not at home for 2‐3 days due to the nature of my work. It is very difficult for me to go to the PHC or even call the counsellor at home. I am always on the road so cannot talk over phone.” (Male, 30 years)

However, a number of key barriers were encountered and strategies were modified to address these (summarized in Table 2 below). Some of the major barriers included men with harmful drinking not seeking health care for their drinking problems, patients and family members not being accustomed to receiving “talking treatments,” expectations about being prescribed medications for the drinking problem, difficulty experienced by patients in adjusting to a collaborative MI stance in a cultural setting where prescriptive advice from health professionals is a norm, difficulties experienced by the counselors to achieve optimal competence to deliver MI, the complexity of practical personal/social problems experienced by patients with harmful drinking, high dropout rates because of practical barriers to attending treatment sessions, and resistance of family members to the treatment.

**Table 2:** Barriers to Delivery of CAP and Adaptations Made to Overcome Them

Barrier	Adaptation
Treatment engagement hindered as primary care attenders rarely seek health care for their harmful drinking, and patients and family members are not accustomed to receiving “talking treatments” and express a desire for medications to treat the drinking problem	Psychoeducation component was modified to provide more comprehensive information to respond to these needs
MI stance was not an acceptable approach in a setting where patients expect prescriptive advice from health professionals	Specific mandatory tasks (e.g., personalized feedback) were prescribed to be achieved by the counselor in the first session with the expectation that a patient would be better able to deal with the MI stance once engaged with the treatment
Challenging for lay counselors to achieve the standards of competence to deliver MI	An accompanying counseling relationship (CR) manual was developed to train counselors in engaging patients and in a wider range of “nonspecific” skills which are essential to deliver MI
Patients with harmful drinking experienced a variety of practical personal/social problems such as difficulty in expressing emotions, which need to be addressed to improve drinking‐related outcomes	Specific modules in CAP were emphasized to tackle these issues, for example, problem‐solving skills
Patients often did not have time for the first session (45 to 60 minutes) after screening positive for harmful drinking	“Abbreviated” first session developed to enable the counselor to commence the engagement process
Dropout rates were high due to practical barriers such as lack of time to attend counseling because of work commitments and inability to travel to the PHC for financial reasons	After the first session in the PHC, home‐based delivery was offered for follow‐up sessions
Although CAP emphasized family involvement, family members sometimes saw counseling as a “waste of time” or patients were unwilling to involve family members	To make an effort to involve a family member in at least 1 session to get a more rounded picture of the problem and further involvement made at the discretion of the counselor and the patient
A third of the patients screening positive for harmful drinking were alcohol dependent	Two major changes made to accommodate this patient group, viz. a structured pathway for referral to medically assisted detoxification to supplement CAP, and provision of information about medications for alcohol dependence

#### Evaluating the Impact of CAP: Pilot Randomized Trial

The emerging version of CAP was evaluated in a pilot RCT conducted over 5 months (participants recruited between August 2013 and October 2013) in 8 PHC in Goa, India. Men aged above 18 years, resident in Goa, and without a comorbid physical or mental illness requiring emergency treatment were eligible for screening with the AUDIT. Those scoring 12 and above on the AUDIT were invited to participate in the trial and were enrolled after obtaining written informed consent. Consenting participants were randomly assigned to CAP or enhanced usual care (EUC). EUC comprised giving the mhGAP treatment guidelines and AUDIT scores of the screened patients to the PHC doctor.

At the end of the internship, 12 lay counselors (10 female), who achieved competence as assessed by standardized role plays, were selected for the pilot RCT. During the pilot study, group supervision was led by a peer lay counselor rather than an expert although an expert always attended and was available if needed (Singla et al., 2014). On average, lay counselors were 25.9 years of age with 15 years of education.

CAP was delivered in 3 phases. In the initial phase, the counselor helped the patient understand the problems his drinking were causing and raised the issue that he may need to change. This was done through a detailed assessment followed by personalized feedback. This was then used to facilitate a commitment to change from the patient which in turn was used to generate a change and action plan that summarized what the patient wanted to do to change his drinking and its related problems, and the actual actions that the patient would take to achieve this goal. In the middle phase, the counselor helped the patient to develop thinking and behavioral skills and techniques (drink refusal, handling the urge to drink, problem solving, and handling difficult emotions) which would allow the patient to make the changes that he desired. In the ending phase, the patient learned how to manage potential or actual relapses using these thinking and behavioral skills and techniques. As this was a pilot trial aimed primarily to inform the final revisions to enhance the acceptability and feasibility of CAP, inform procedures for the definitive RCT (e.g., screening procedures, recruitment rates), and generate preliminary estimates of impact, sample size estimations were not carried out. The primary outcome measures, assessed 2 months postenrollment, were the Alcohol Timeline Followback (TLFB) (Sobell and Sobell, 1992) assessing the quantity of drinking in the past 2 weeks; the AUDIT; and the Short Inventory of Problems (SIP), a 15‐item questionnaire that measures physical, social, intrapersonal, impulsive, and interpersonal consequences of alcohol consumption (Alterman et al., 2009). Intention‐to‐treat analyses adjusted for baseline AUDIT score as well as clustering at the level of the PHC were carried out using Stata 11 (StataCorp LP, College Station, TX). During analyses, missing observations were dropped in the statistical models.

The sample (Table 3) was comprised of 53 men who agreed to participate from 118 eligible screened patients (participation rate 44.9%). Twenty seven were assigned to CAP and 26 to EUC. Forty‐seven (88.7%) participants completed the outcome assessment. In total, 53 sessions were delivered with a mean (SD) of 2.1 (0.9). Thirteen (48.2%) patients completed treatment and the rest dropped out. There were no significant differences between those who completed follow‐up and those who did not, with regard to age, marital, educational, or occupational status; baseline AUDIT score; or allocation status. There were no statistically significant differences between those allocated to CAP or EUC, with regard to age, marital, educational, or occupational status; and baseline AUDIT. The amount of alcohol consumed in the past 2 weeks, mean AUDIT score, and alcohol‐related problems were all lower in the CAP arm compared to the EUC arm, but the between‐group adjusted mean differences were not statistically significant (Table 4). There were nonsignificant reductions in outcomes in participants who completed treatment compared with those who dropped out with regard to mean AUDIT (8.6 vs. 12.9, *p* = 0.3), mean alcohol (gms) consumed in past 2 weeks (272.5 vs. 368.4, *p* = 0.6), and mean SIP score (6.4 vs. 8, *p* = 0.7).

**Table 3:** Baseline Characteristics of Participants in Pilot Trial

Variable	Counseling for Alcohol Problems (*n* = 27)	Enhanced Usual Care (*n* = 26)
Mean age in years (SD)	41.4 (11.4)	41.9 (11.3)
Married (%)	15 (79.0) mv = 3	20 (83.3) mv = 7
Completed at least primary education (%)	19 (79.2) mv = 3	17 (89.5) mv = 7
Employed (%)	18 (75.0) mv = 3	17 (89.5) mv = 7
Mean AUDIT score (SD)	18.5 (7.2)	20.8 (6.6)

mv, missing values.

**Table 4:** Effect of CAP on Drinking and Other Outcomes

Outcome	Counseling for Alcohol Problems (*n* = 23)	Enhanced Usual Care (*n* = 24)	Adjusted mean difference (95%CI)^a^	*p*‐Value
AUDIT score	10.5 (10.0)	16.9 (8.3)	−4.9 (−10.1, 0.2)	0.06
Mean alcohol (gms) consumed in past 2 weeks	311.8 (541.6)	548.4 (681.7)	−145.8 (−526.4, 234.9)	0.4
Mean Short Inventory of Problems score	7.1 (11.7)	14.8 (15.3)	−4.7 (−11.8, 2.4)	0.19

a Adjusted for baseline AUDIT score and primary healthcare centers.

## Discussion

This paper describes the process of development of CAP, a manualized brief PT for harmful drinking delivered by lay counselors in primary care settings in India. In doing so, we have described a systematic methodology in settings which differ quite dramatically in several respects from those in high‐income countries (HIC): the vast gap in mental health human resources; vast differences in mental health “literacy”; lack of substantial “local” knowledge on nonpharmacological methods for addressing mental health problems; and the heavy reliance of primary healthcare programs on lay health workers who, realistically, have no equivalent in HIC.

The treatment development process started with mapping the evidence base for effectiveness of PT for AUD in primary care. This was supplemented with contextual evidence by describing the explanatory models/causal attributions; coping strategies used by patients with AUD and their family members; and treatment strategies used by mental health professionals in South Asia in general and India in particular. The classes of PT were dismantled into their component strategies and assessed by mental health professionals and lay counselors for contextual acceptability, feasibility, and safety for delivery by lay counselors. Strategies with evidence base for effectiveness, were contextually appropriate, and were perceived to be acceptable, feasible, and safe to be delivered by lay counselors were assembled into a PT in Stage 2. This PT was then iteratively refined, modified, and tested through a clinical case series and pilot study. The primary goal of the final steps was to evaluate and refine the acceptability and feasibility of the delivery of CAP; a secondary goal was to generate preliminary evidence of its effectiveness. The final pilot RCT showed a higher treatment completion rate than the case series and trends indicating improvements on all 3 primary outcomes compared with EUC.

Based on the diversity of the severity of AUD and patient needs, CAP was delivered in 1 to 4 sessions; our experience showed that the initial phase was typically completed in the first session, the middle phase in the second session, and the ending phase in sessions 3 or 4. However, CAP has flexibility on what can be done in individual sessions and the focus is on achieving the goals of the particular phase. For example, with some patients, CAP may move faster, and the initial and middle phases may be covered in 1 session, while with other patients, CAP may move more slowly, and it may take 2 sessions to work through the initial phase, and the remaining 2 sessions may be needed to work though the remaining phases. The sessions are conducted on a weekly or fortnightly basis in the patient's home or the PHC, as per the patient's convenience. For patients with alcohol dependence, in addition to the PT, the patient also gets a structured referral for medically assisted detoxification. Any patient who achieves treatment goals is discharged regardless of number of sessions completed, as is any patient who completes the maximum of 4 sessions. The CAP manual can be downloaded at: http://www.sangath.com/images/manuals/Counselling%20for%20Alcohol%20Problems_Manual.pdf.

The CAP treatment development experience demonstrates how contextual factors can substantively influence the content and delivery format of a PT. Although the manual development began with the Project MATCH MET manual, the final CAP that emerged took on a substantially different form. For example, unlike in Project MATCH in which the treatment was provided to patients specifically seeking help from an alcohol service, the vast majority of patients with harmful drinking in primary care are not specifically seeking help for their drinking problem. Further, many patients have limited literacy. Thus, CAP employs comprehensive and pictorially dominated psychoeducational materials to engage patients in the treatment. Similarly, an intervention based on “pure” MET relying solely on developing the motivation to change is often unacceptable to patients not used to “talking treatments,” especially nondirective ones. This led to the addition of skills‐based modules to the treatment, for example, drink refusal skills. Numerous practical barriers affect the accessibility of PT in primary care settings, such as the likely loss of wages if the patient attends counseling during working hours. These needed to be addressed through adaptations such as home‐based delivery. Incorporation of such contextually appropriate strategies is as important as the strategies from empirically supported PT to enhance contextual feasibility and acceptability of the PT. These also help create a plausible theoretical framework for the PT.

On the other hand, our treatment development process highlights that despite explicit attention to contextually appropriate coping strategies and explanatory models/causal attributions, most strategies in the final treatment package appeared to have commonalities with other treatment packages used in HIC contexts. Significant structural and cultural barriers reduce treatment engagement and need to be addressed proactively to enhance treatment adherence. Following a systematic process of treatment development enhances the likelihood of acceptability and feasibility of lay counselor‐delivered PTs. For example, through the case series, we realized that one of the major barriers to treatment completion was delivering CAP only in the PHC. Consequently, we introduced home delivery as an option, and this led to reduced treatment dropouts, indicating an enhancement of treatment acceptability.

The only comparable effort to develop a PT for alcohol problems in an LMIC was an effort to adapt CBT for alcohol use among HIV‐infected outpatients in Kenya (Papas et al., 2011). This study had several similarities with PREMIUM, for example, the substantial time spent on the development of the treatment; triangulation of data collected using mixed methods from diverse stakeholders; careful consideration of issues related to recruitment, training, competency evaluation, and maintenance of the counselors; and iterative use of data from the pilot study to refine the treatment. A key difference was that while Papas and colleagues. (2011) started with an “off the shelf” treatment developed in Western settings, PREMIUM dismantled evidence‐based psychosocial treatments into their component strategies and added contextually appropriate strategies to create a pool of strategies which formed the basis for a new treatment.

The pilot RCT has showed that the PT has promising preliminary evidence of impact, though not statistically significant due to the fact that the trial was not powered to detect a significant effect. A definitive evaluation of the effectiveness and cost‐effectiveness of the CAP is currently being carried out through an RCT in Goa, with a target sample size of 400 patients (Trial registration ISRCTN76465238) (Patel et al., 2014). The expectation is that, in a fully powered trial with experienced counselors and enhanced emphasis on treatment completion, we will observe a statistically significant effect of the intervention. Another limitation was the low treatment completion rates. This finding informed the procedures to improve retention in treatment, for example, SMS sent to patients reminding them of appointments. Other limitations of the study were the limited generalizability as the study was conducted only with males and only in 2 sites.

The main strength of this study is the structured methodology that has been used to address the challenges inherent to the development, evaluation, and implementation of psychosocial interventions in low resource and culturally diverse contexts, which in turn has led to an intervention which is acceptable to various stakeholders, feasible to deliver, and hence has greater chances of being effective and scalable. If the resulting intervention is found to be cost‐effective, then this has major implications for alcohol treatments in low resource settings. In LMIC, the gap between those who could benefit from mental health interventions and those who receive such care is very large, in particular for AUD (Saxena et al., 2007; WHO World Mental Health Survey Consortium, 2004). A contextually adapted intervention like CAP which can be delivered by nonspecialists has the potential of being scaled up in low resource settings, thus helping reduce the treatment gap.

## Supporting information


**Table S1.** Criteria for selection of psychological treatments.
**Appendix S1.** Search strategies for international and regional reviews.
**Appendix S2.** Key informants and libraries used in the literature reviews.
