# Supplementary material for: The Systematic Development and Pilot Randomized Evaluation of Counselling for Alcohol Problems, a Lay Counselor‐Delivered Psychological Treatment for Harmful Drinking in Primary Care in India: The PREMIUM Study
Source: Alcohol Clin Exp Res. 2015 Feb 19;39(3):522–31. doi: 10.1111/acer.12653 (PMC4862032; doi:10.1111/acer.12653)
Supplement: Supplementary file 1 — Table S1. Criteria for selection of psychological treatments. Appendix S1. Search strategies for international and regional reviews. Appendix S2. Key informants and libraries used in the literature reviews. [file ACER-39-522-s001.docx]

**SUPPLEMENTARY MATERIAL FOR ONLINE PUBLICATION**

Online Table 1: Criteria for selection of Psychological Treatments

| **Evidence for Effectiveness** | **Rating** | **Generalisability to the setting**  **(1 point for each criteria)** |
| --- | --- | --- |
| Systematic review | 4 | - Evidence from LMIC - Evidence in PHC setting - Evidence of delivery by lay counsellors - Evidence in a sample of ethnic minority/disadvantaged population. |
| More than one RCT | 3 |  |
| One RCT | 2 |  |
| Only observational evidence | 1 |  |

ONLINE APPENDIX S1

**Search strategies**

a) International review: We searched Medline and PsycINFO electronic databases using the following search terms in any field: (Alcohol OR drinking OR addiction) AND (Psycho* OR therapy OR counseling OR treatment) NOT (pharma* OR medic*). The eligibility criteria were as follows: We included systematic reviews, meta-analyses and randomized controlled trials (RCT) published from 1st January 2009 to 31st March 2011 (as the mhGap reviews involved a systematic literature search on PTs prior to 2009), studies in adults (19 years and above), studies involving patients with AUD including those with comorbid mental disorders and studies testing any non-pharmacological treatments with the exception of studies of computerized/internet based treatments. We excluded studies conducted exclusively in hazardous drinkers, exclusively female samples (PREMIUM focused on treatment for men as the prevalence of AUD among women in India is very low), pilot studies and treatment development studies. There was no restriction on language, sample size, type of comparison group or outcome measure.

b) Regional review: We searched Medline, IndMed (indexing articles published in Indian biomedical journals), PsycINFO and PsycExtra from 1st January 1990 to 31st March 2011. For Medline, PsycINFO and PsycExtra we used the following search terms: (Alcohol OR drinking OR addiction) AND (South Asia OR India OR Pakistan OR Bangladesh OR Sri Lanka OR Bhutan OR Nepal OR Maldives OR Afghanistan) AND (Psycho* OR therapy OR counseling OR behavior OR intervention OR harm reduction) NOT (pharma OR medic*). As the IndMed search engine allows use of maximum six words at a time we conducted two searches as follows: Search 1- alcohol OR drinking OR addiction OR counseling OR psycho* OR therapy; Search 2-alcohol OR drinking OR addiction OR behavior OR intervention OR harm reduction. The eligibility criteria was as follows: We included published reports of any type of studies, systematic reviews, narrative reviews, case reports and treatment manuals describing non pharmacological treatments for AUD in South Asia. We included studies in adults (19 years and above) with any type of AUD or AUD with co morbidity. We also searched grey literature by hand searching table of contents of non indexed journals and, searching library (Online appendix B) catalogues for books, project reports, manuals and dissertations, and obtaining a list of relevant literature from key informants (Online appendix B)

**ONLINE APPENDIX S2:**

**List of regional key informants for regional AUD literature search**

1. Pratima Murthy, Chief, Deaddiction Unit, NIMHANS, Bangalore, India
2. Vivek Benegal, Additional Professor, Deaddiction Unit, NIMHANS, Bangalore, India
3. Shanthi Ranganathan, Director, TT Krishnammachari Institute of Deaddiction, Chennai, India
4. Shubhangi Parkar, Professor of Psychiatry, KEM, Mumbai, India
5. Mohan Isaac, Ex-Professor of Psychiatry, NIMHANS, Bangalore, India
6. Vasudeo Parulekar, Head, Department of Psychiatry, KEM Hospital, Pune, India
7. Madhabika Nayak, Associate Scientist, Alcohol Research Group, CA, USA/India
8. Mark Jordans, Senior Research & Technical Advisor, HealthNet TPO, Nepal/Netherlands
9. Aravind Pillai, former coordinator of alcohol research program, Sangath, India.

**List of libraries in India visited for AUD literature search**

1. Tata Institute of Social Sciences, Mumbai
2. Post Graduate Institute of Medical Education and Research, Chandigarh
3. National Institute of Mental Health and Neurosciences, Bangalore
4. TTK Institute of Deaddiction, Chennai
5. Institute of Human Behavior & Allied Sciences, New Delhi
6. National Library of Medicine, New Delhi
7. National Drug Dependence and Treatment Centre, New Delhi
